# Guided internet-based cognitive behavioral therapy for obsessive-compulsive disorder: A multicenter randomized controlled trial in Japan

**DOI:** 10.1016/j.invent.2022.100515

**Published:** 2022-02-24

**Authors:** Kazuki Matsumoto, Sayo Hamatani, Takuya Makino, Jumpei Takahashi, Futoshi Suzuki, Tomoko Ida, Shoko Hamamura, Shinichiro Takiguchi, Akemi Tomoda, Ichiro M. Omori, Hirotaka Kosaka, Seina Shinno, Tomoki Ikai, Hiroyuki Hayashi, Hiroto Katayama, Yuki Shiko, Yoshihito Ozawa, Yohei Kawasaki, Chihiro Sutoh, Eiji Shimizu

**Affiliations:** a Research Center for Child Mental Development, Chiba University, Chiba 260-8588, Japan; b Laboratory of Neuropsychology, Institute of Liberal Arts and Science, Kanazawa University, Ishikawa 920-1192, Japan; c Research Center for Child Mental Development, University of Fukui, Fukui 910-1192, Japan; d Department of Child Development, United Graduate School of Child Development, University of Fukui, Fukui 910-1193, Japan; e Department of Neuropsychiatry, University of Fukui, Fukui 910-1192, Japan; f Department of Integrated Medical Sciences, Graduate School of Medicine, University of Fukui, Fukui 910-1192, Japan; g Department of Integrated Advanced Medicine, Graduate School of Medicine, University of Fukui, Fukui 910-1192, Japan; h Department of Primary Health Care, Faculty of Medicine, University of Fukui, Fukui 910-1192, Japan; i Department of Emergency and General Medicine, University of Fukui Hospital, Fukui 910-1192, Japan; j Kokorotokarada Clinic Fukui, Fukui 910-0067, Japan; k Biostatistics Section Clinical Research Center, Chiba University Hospital, Chiba 260-8677, Japan; l Faculty of Nursing, Japanese Red Cross College of Nursing, Tokyo 150-0012, Japan; m Department of Cognitive Behavioral Physiology, Graduate School of Medicine, Chiba University, Chiba 260-8588, Japan; n Cognitive Behavioral Therapy Center, Chiba University Hospital, Chiba 260-8588, Japan

**Keywords:** ERP, Exposure and Response Prevention, ICBT, internet-based cognitive behavioral therapy, JART, Japanese adult rating test, OCI, obsessive compulsive inventory, PRT, progressive relaxation therapy, TAU, treatment as usual, Y-BOCS, Yale-Brown obsessive-compulsive scale, Cost-effectiveness, Internet-based cognitive behavior therapy, Randomized controlled trial, Obsessive-compulsive disroder, Treatment as usual

## Abstract

Few studies have compared the effectiveness of internet-based cognitive behavior therapy (ICBT) for obsessive-compulsive disorder (OCD) with treatment as usual (TAU). We investigated the effectiveness of guided ICBT for patients with OCD. This prospective, randomized, controlled, assessor-blinded, multicenter clinical trial was conducted at three facilities in Japan from January 2020 to March 2021. Thirty-one patients with OCD as the primary diagnosis participated in the trial and were randomly assigned to either the intervention group or the control group. The primary outcome was the Yale–Brown obsessive-compulsive scale score; the assessors were blinded. Results of the analysis of covariance among the groups were significantly different between the groups (*p* < 0.01, effect size Cohen's *d* = 1.05), indicating the superiority of guided ICBT. The results suggest that guided ICBT is more effective than TAU for treating OCD.

**RCT registration:**

UMIN Clinical Trials Registry (UMIN000039375).

## Highlights

• A multi-center randomized controlled trial was conducted in Japan.
• Participants were allocated to either the Internet-based CBT (ICBT) group or the treatment as usual (TAU) group.
• Results of this study show the effectiveness of guided ICBT was superior to TAU.
• Guided ICBT for OCD may be cost-effective in Japan.

## 1 Introduction

The effectiveness of cognitive behavioral therapy (CBT) in treating obsessive-compulsive disorder (OCD) has been demonstrated in face-to-face sessions (Skapinakis et al., 2016). Poor availability of CBT, the first-line treatment of choice for OCD in multiple treatment guidelines (National Institute for Health and Excellence: NICE, 2005; Wheaton et al., 2016), has been a serious issue. Very few people receive CBT because of lack of treatment resources or physical barriers (Cavanagh, 2014; O'Neill and Feusner, 2015). Previous studies have reported that only 5–6.2% of patients with OCD find CBT accessible (Blanco et al., 2006; Takahashi et al., 2018; Torres et al., 2007). As a solution, internet-based CBT (ICBT) has been developed to overcome geographical barriers and lack of treatment resources.

Equal effectiveness of ICBT and face-to-face CBT for anxiety disorders was suggested through meta-analyses (Andersson et al., 2014; Carlbring et al., 2018). However, these meta-analyses included no randomized controlled trials (RCT) targeting patients with OCD. Although previous RCTs showed the effectiveness, only two compared guided ICBT for patients with OCD to treatment as usual (TAU) or wait-list control (Herbst et al., 2014; Mahoney et al., 2014). In other RCTs, ICBT was compared to attention control (Andersson et al., 2012) and progressive relaxation therapy (PRT) (Kyrios et al., 2018). Hence, investigating the effectiveness of guided ICBT when combined with TAU for OCD could provide valuable information for patients with OCD, clinicians, and health policy makers, for decision makers regarding the introduction of guided ICBT.

ICBT for OCD was recently suggested to be more cost-effective than face-to-face CBT and PRT (Osborne et al., 2019). Cost-effectiveness analysis of interventions depends on the medical infrastructure of each country. However, cost-effectiveness of guided ICBT has not been analyzed in the Japanese context. Furthermore, little is known about the therapeutic predictors of guided ICBT (Andersson and Titov, 2014; Kyrios et al., 2018).

The objectives of the present study are: 1) to investigate the effectiveness of guided ICBT with TAU; 2) to evaluate cost-effectiveness of the intervention in Japan; and 3) to identify the characteristics of patients who responded.

## 2 Material and methods

The current RCT has been registered in the Japanese clinical trial registration database (UMIN000039375). This study was approved by the Institutional Review Board of Chiba University Hospital (G2019017). The protocol of the RCT was previously published (Matsumoto et al., 2020a: see Supplementary File Table S1 for protocol changes and reasons). Herein, we report our findings from the RCT, adhering to the CONSORT 2010 statement (Schulz et al., 2010; see the Supplementary File Table S2).

### 2.1 Design

The prospective, randomized controlled, open-blind, multicenter trial was conducted from January 2020 to March 2021. The three included facilities were the Chiba University Hospital, University of Fukui Hospital, and Kokoro to Karada Clinic Fukui. The sample size of the RCT was calculated by using the statistical analysis software G*power 3.1 (Faul et al., 2007; Faul et al., 2009). The predicted effect size was at least 1.00, based on two previous studies comparing guided ICBT with an active control (Andersson et al., 2012; Mahoney et al., 2014). The directionality of the test was set as two-sided, significance level at 0.05%, and the power (1 − β) was 80%. According to the sample size, the RCT needed a minimum of 14 participants per group. Considering a 10% dropout rate, the final sample size was set as 32.

### 2.2 Participants

The eligibility criteria were: (1) a diagnosis of OCD, (2) aged 15–60 years, (3) total score ≥ 14 on the Yale–Brown obsessive-compulsive scale (Y–BOCS) (Goodman et al., 1989; Hamagaki et al., 1999), and (4) sufficient information and communication technology (ICT) literacy to undertake e-learning. Exclusion criteria were: (1) diagnosis of schizophrenia, dementia, antisocial personality disorder, (2) a history of suicide attempts and substance use in the last 12 months, (3) experience with CBT including exposure therapy within the past two years, and (4) presence of a progressive illness such as cancer.

### 2.3 Recruitment

**Fig. 1:**
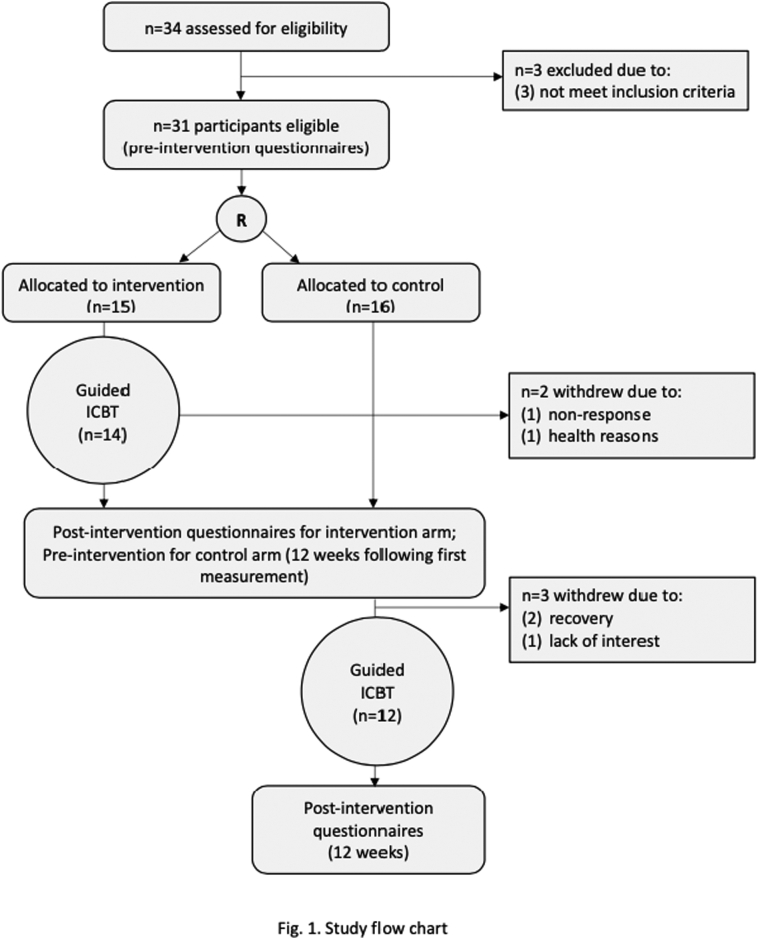
Study flow chart.

Fig. 1 shows the flow chart of the study. The participants were recruited from January to December 2020 via distributed leaflets at each hospital, the website of Chiba University, and the recruitment service of SOKEN, Inc. Patients in need of guided ICBT accessed this RCT information on websites of Research Center for Child Mental Development, Chiba University and applied for RCT participation. The details of the research contents were explained to them through telephone; subsequently, those willing to participate gave informed consent in-person at the nearest facilities near their area of residence. In case of minor participants (15–19 years old), parental written informed consent was obtained.

### 2.4 Eligibility assessment

The eligibility assessment was performed by researchers in a single face-to-face session. The researchers assessed the eligibility of applicants by interviewing them about: sex, age, years of education, age of onset, comorbidities, medication content (pharmacotherapy), and estimated intelligence quotient (IQ) using the Japanese adult rating test (JART) (Matsuoka et al., 2006; Nelson and Willison, 1991). OCD and other psychiatric disorders were evaluated by using the Mini-International Neuropsychiatric Interview (Muramatsu et al., 2007; Sheehan et al., 1998).

### 2.5 Randomization

Participants were allocated to either the intervention group (guided ICBT + TAU) or control group (TAU + waiting list to intervention) in a 1:1 ratio using the minimization method, based on sex (male, female, or other) and the severity of obsessive-compulsive symptoms measured by Y-BOCS (total score more or less than 20) at baseline (Goodman et al., 1989; Hamagaki et al., 1999). The randomization procedure was conducted by the independent data management team of the Chiba University Hospital using a computer program. Participants allocated to the control group were informed that they could receive the ICBT program after this RCT was completed. Specifically, we conducted a rescue trial for the participants assigned to the control group (described summary of this rescue trial on Supplementary File).

### 2.6 Interventions

The ICBT program, was developed by the first author (KM) on the e-learning platform (LearningBox®) of Tatsuno System Inc. Feasibility of the treatment has been demonstrated in a cases series involving Japanese patients with OCD (Matsumoto et al., 2020b). The ICBT program consisted of 12 modules; each module included explanations and cognitive-behavioral training techniques for changing cognitive-behavioral patterns in patients with OCD (Table 1). Completion of each module was estimated to take 10–30 min. The participants were encouraged to attend one module per week. Two clinical psychologists with a PhD (one male [KM] and female [SH] in their early thirties) who completed the training in CBT and had experience providing CBT for OCD guided the participants using Share Medical, Co.'s chat tool (MediLine®). The quality of the ICBT was controlled by a senior supervisor (ES). The control group was provided with TAU, and the condition of the participants was controlled by their psychiatrists. The participants of the control group could receive the intervention after the RCT. In principle, alterations to medication were restricted, and none of the participants received alterations to their medication during the RCT.

**Table 1:** Modules and homework each session in the ICBT.

#	Module	Homework
1	Psychoeducation, case-formulation	Creating a figure of case-formulation
2	Therapeutic goal setting, anxiety/exposure hierarchy	Creating an anxiety/exposure hierarchy
3	Typical beliefs in patients with OCD, theory AB exercise	Describe Theory AB
4	Behavioral experiment to verify the beliefs of each subtype: contamination; harm; symmetry, unacceptable or taboo thoughts.	Implement a behavioral experiment
5	Exposure and Response Prevention (ERP) I	Implement ERP on a relatively low target of the anxiety/exposure hierarchy
6	ERP II, stop thought suppression	Implement ERP on a relatively low target of the anxiety/exposure hierarchy, notice and stop thought suppression
7	ERP III, breathing exercise for relaxation	Implement ERP on targets of greater fear; relaxation instead of avoidance to continue ERP
8	ERP IV	Implement ERP on targets of greater fear; conduct daily ERP
9	ERP V	Implement ERP on targets of greater fear; conduct daily ERP
10	ERP VI and tape exposure	Implement ERP on targets of greater fear repeatedly and tape exposure to fear-causing intrusive thoughts
11	ERP VII, relaxation, and tape exposure	Implement ERP on targets of greater fear, conduct daily ERP, and evaluate the achievement of therapeutic goals
12	Prevention of relapse	Create a prevention of relapse sheet

ERP, Exposure and Response Prevention; ICBT, internet-based cognitive behavioral therapy.

### 2.7 Primary outcome

The primary outcome was differences in symptoms of OCD at 0 week (baseline) and 12 weeks (post-intervention), measured by the Y-BOCS (Goodman et al., 1989; Hamagaki et al., 1999; 10 items; range 0–40). Three independent assessors blinded to the allocation assessed the Y-BOCS scores in face-to-face or telephone interviews. We adopted the following definitions of treatment response and remission: treatment response, >35% reduction of the total Y-BOCS score from baseline to post-intervention; remission, the total Y-BOCS score ≤ 13 at post-intervention (Farris et al., 2013).

### 2.8 Secondary outcomes

Unless otherwise specified, all secondary outcomes were measured at the time of screening and immediately post-intervention.

#### 2.8.1 Mental health

Secondary mental health outcomes included symptoms of OCD as measured by the obsessive-compulsive inventory (OCI) (Foa et al., 1998; Ishikawa et al., 2014; 42 items, range 0–168), depression as measured by the patient health questionnaire 9 items (PHQ-9) (Muramatsu, 2014; Spitzer et al., 1999; 9 items, range 0–27), and generalized anxiety as measured by the generalized anxiety disorder 7 items (GAD-7) (Muramatsu, 2014; Spitzer et al., 2006; 7 items, range 0–21).

#### 2.8.2 Quality of Life

The quality of life (QOL) was measured using the EuroQol 5 dimension-5 List (EQ-5D-5L) (van Hout et al., 2012; Shiroiwa et al., 2016; 5 times, range 0–1). The EQ-5D-5L, developed by EuroQoL group based on EuroQol 5 dimension-3 level (EuroQol Group, 1990; Tsuchiya et al., 2002), can measure a generic preference-based health. The EQ-5D can provide QOL values for use in calculating quality-adjusted life years (QALYs) in the economic assessment of medical technology (Ikeda et al., 2015). In the calculated QOL, zero represents death and 1.0 represents complete health.

#### 2.8.3 Additional measure

Therapeutic relationships built with ICBT guided using chat tools as measured by the working alliance inventory-short form (WAI-SF) (Hatcher and Gillaspy, 2007; 12 items, 12–84). WAI-SF was evaluated only at 12 weeks.

### 2.9 Statistical analyses

#### 2.9.1 Effectiveness evaluation

We tested if TAU + intervention was superior to TAU + waiting list to intervention in terms of effects on participants' obsessive-compulsive symptoms severity and secondary outcomes from baseline to post-intervention. We did not complement in the missing values, and data from participants who had never conducted the ICBT in the intervention group were excluded from the effectiveness analysis. A significance level of 0.05 (two-sided) was used for all analyses. Analyses were conducted with the SAS® 9.4 software (SAS Institute Inc., North Carolina, USA).

Differences in effects between the two study conditions were assessed using analysis of covariance (ANCOVA). The estimated IQ, use of antidepressants such as selective serotonin reuptake inhibitor (SSRI), and baseline total Y-BOCS scores were used as the covariate. We calculate the between-group standardized mean difference (viz. Cohen's *d*) as an effect size. For the primary outcome, we also calculated the within-group effect sizes for both the groups. For interpretation of effect size, we adapted Cohen's (1988) criteria. Cohen's *d* = 0.2 can be considered a small effect, Cohen's *d* = 0.5 a medium, and Cohen's *d* = 0.8 a large effect.

We also compared the proportion of participants with reliable response at reduction in total score ≥ 35% and reliable remission at a score ≤ 13 in Y-BOCS (Farris et al., 2013). We compared the proportions of responders and remissioners in the intervention group and the control group at post-treatment using the Fisher's exact test.

#### 2.9.2 Assessment of blinding

The three independent assessors did not contact the participants during the RCT for purposes other than the assessment. The success of blinding was assessed using the Bang method (Bang et al., 2004). At week 12, after collecting data on post-treatment outcomes, the independent assessors were asked “What type of treatment do you think the participant received?” They answered “guided ICBT,” “TAU” or “I do not know”. Bang's blinding index ranges between −1 and 1, with 0 as a null value indicating complete blinding, 1 representing complete unblinding, and −1 representing all participants guess their treatment allocation incorrectly. Therefore, when one-sided CI did not cover the 0 value, the study was regarded as lacking blinding (Bang et al., 2004).

#### 2.9.3 Cost-effectiveness evaluation

We calculated cost-effectiveness analyses (CEA) models to evaluate cost-effectiveness for the guided ICBT for patients with OCD by the three incremental cost-effectiveness ratios (ICER).

First, the cost required for an additional 1 QALY was calculated using the following formula (NICE, 2013; Siebert et al., 2012):

$$\mathrm{ICER}=\frac{\mathrm{\Delta Cos}t}{\Delta \mathrm{QALY}}$$

As the ICBT group also received TAU in this study, the increased intervention cost was considered the cost of the guided ICBT. The total therapist-guided ICBT cost was calculated using the healthcare costs (JP¥3500 × 12 sessions/patient) and costs of the ICBT program (the e-learning platform (LearningBox) JP¥5500 per month × 3-month and chat tool (MediLine) ¥6600 per month × 3-month (Ministry of Health, Labor and Welfare, 2020). ΔQALY was calculated by the difference between each group at post-treatment. In addition, the willingness-to-pay (WTP) for one additional QALY was set at JP¥ 5 million (USD 47,619) (Fukuda and Shiroiwa, 2019).

Second, the cost required to increase a treatment responder by one was calculated using the following formula:

$$\mathrm{ICER}=\frac{\mathrm{\Delta Cos}t}{\Delta \mathrm{Responder}}$$

ΔCost was calculated by the above method. ΔResponders were calculated from the differences in each group according to the Y-BOCS criteria: the total score ≥ 35% (Farris et al., 2013).

Third, the cost required to increase a patient with remission by one was calculated using the following formula:

$$\mathrm{ICER}=\frac{\Delta \mathrm{Cos}t}{\mathrm{\Delta Remission}}$$

ΔCost was calculated by the above method. ΔRemission were calculated from the differences in each group according to the Y-BOCS criteria of total score ≤ 13 (Farris et al., 2013).

#### 2.9.4 Secondary analysis in participants completed the ICBT

We performed a series of multiple regression analyses to investigate the predictors of OCD symptomatology improvement. The treatment response rate for total Y-BOCS was set as the dependent variable. The independent variables were pharmacotherapy, depressive symptoms, general anxiety, and estimated IQ. Variables were entered for analysis in a multivariate model with a step-by-step procedure of forward selection (*F* < 0.05, *F* ≥ 0.10 for exclusion). The population analyzed was participants, who performed at least one ICBT module, in this RCT and a rescue trial. Multicollinearity was measured using the variance expansion factor (VIF) and margin of error. If the VIF value was >4.0 or the margin of error was >0.2, multicollinearity was considered a problem (Hair et al., 2010).

## 3 Results

In the intervention group, a participant dropped out before the first session and could not contact further. A control group participant dropped out because of a serious adverse event (detailed under Section 3.6 adverse events). Therefore, data of the thirty participants who were assessed for all the outcomes at all the time points were analyzed. Demographic data of the participants are summarized in Table 2. Characteristic of participants allocated the intervention and control groups were largely similar. Descriptive data for all outcomes is shown in Table 3.

**Table 2:** Comparisons between the intervention and the control groups at baseline.

	Intervention (n = 14)	Control (n = 16)	*p*-Value
Sex (female), n	8 (57.1%)	9 (56.3%)	0.404
Age, mean (SD)	31.6 (14.0)	28.7 (11.3)	0.537
Years of education, mean (SD)	13.4 (2.9)	12.8 (2.1)	0.509
Estimated IQ (JART) mean (SD)	102.0 (12.4)	100.7 (6.6)	0.715
Obsessive-compulsive symptoms (Y-BOCS), mean (SD)	22.5 (4.2)	24.3 (6.3)	0.366
Depression (PHQ-9), mean (SD)	9.7 (6.5)	9.0 (6.1)	0.759
Generalized Anxiety Disorder (GAD-7), mean (SD)	10.4 (5.6)	9.8 (4.9)	0.779

GAD, generalized anxiety disorder; JART, Japanese adult rating test; PHQ, patient health questionnaire; Y-BOCS, Yale-Brown obsessive-compulsive scale.

**Table 3:** Results of analyses of covariance.

Outcome and assessment point	Intervention (n = 14)		Control (n = 16)		ANCOVA	
	Mean	SD	Mean	SD	*F*	*p*
Y-BOCS (0–40)						
– Pre (baseline)	22.50	4.15	24.31	6.28		
– Post (12 weeks)	14.71	5.65	20.87	7.23	2.03	0.009
OCI (0–168)						
– Pre (baseline)	61.57	28.42	63.13	28.12		
– Post (12 weeks)	47.14	27.76	51.47	28.99	0.72	0.779
PHQ-9 (0–27)						
– Pre (baseline)	9.71	6.52	9.00	6.11		
– Post (12 weeks)	8.64	6.56	9.27	6.67	1.14	0.665
GAD-7 (0−21)						
– Pre (baseline)	10.36	5.61	9.81	4.93		
– Post (12 weeks)	8.36	4.91	8.20	5.05	1.28	0.947
EQ-5D (0.000–1.000)						
– Pre (baseline)	0.6757	0.13	0.7093	0.18		
– Post (12 weeks)	0.7157	0.22	0.7350	0.19	0.40	0.98

EQ, EuroQol; GAD, generalized anxiety disorder; OCI, obsessive compulsive inventory; PHQ, patient health questionnaire; Y-BOCS, Yale-Brown obsessive-compulsive scale.

### 3.1 Effectiveness analyses

#### 3.1.1 Obsessive-compulsive symptoms

**Fig. 2:**
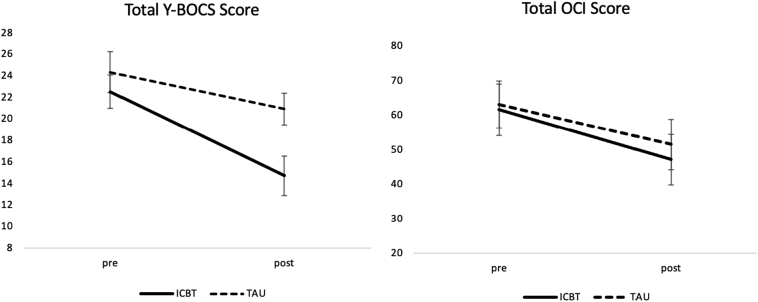
Analysis of obsessive-compulsive symptoms severity scores. The graphic presents mean values and 95% confidence interval.

We observed a decrease in obsessive-compulsive symptoms between pre- and post-intervention in each group (Fig. 2). The total Y-BOCS scores (range, 0–40) decreased within the intervention group from a mean (SD) of 22.5 (4.2) to 14.7 (5.7), with an effect size of Cohen's *d* = 1.61. The control group total Y-BOCS scores mean (SD) also decreased from 24.3 (6.3) to 20.9 (7.2), with an effect size of Cohen's *d* = 0.51. Result of the ANCOVA of the primary outcome indicated a significant difference between group effect on obsessive-compulsive symptoms (*F* = 2.03, *p* = 0.009), favoring the intervention group. A large effect size (Cohen's *d* = 1.05) was found between groups for the primary outcome. The Fisher's exact test revealed that significantly more participants in the intervention group (n = 9) were classified as reliable responders than in the control group (n = 2) at post-intervention (Odds ratio = 10.7, 95% CI: 1.48–134.62, *p* = 0.008). Similar results were shown for reliable remission; participants in the intervention group (n = 7) showed better recovery than in the control group (n = 2) at post-intervention (Odds ratio = 6.06, 95% CI: 0.85–75.26, *p* = 0.0502).

As a secondary outcome of obsessive-compulsive symptoms, the total OCI scores (range, 0–168) decreased within the intervention group from a mean (SD) of 61.6 (28.4) to 47.1 (27.8), and an effect size was Cohen's *d* = 0.52. The control group total OCI scores also decrease from 63.1 (28.1) to 51.5 (29.0), and an effect size was Cohen's *d* = 0.41. Result of the ANCOVA of this secondary outcome indicated a nonsignificant difference between group effect on obsessive-compulsive symptoms (*F* = 0.72, *p* = 0.78).

#### 3.1.2 Depression/generalized anxiety symptoms and QOL

Results of the secondary outcome analyses for depression, generalized anxiety, and QOL, are shown in Table 3. ANCOVAs showed no significant (*p* < 0.05) differences between the intervention and control groups for all the secondary outcomes.

### 3.2 Results of blinding

Indices of blinding of the primary outcome were −0.21 (95% CI: −0.24 to 0.66) in the intervention group and 0.07 (95% CI: −0.37 to 0.50) in the control group, suggesting that blinding might be successful (see the Supplementary File Table S3 for details).

### 3.3 Cost-effectiveness analysis

The calculated cost of the intervention was JP¥ 99,000. The difference of increased QALY was 0.02 between the intervention group and the control group. Thus, ICER was calculated as JP¥ 4,950,000 (US$ 45,000 converted at JP¥ 110 per 1 dollar, €38,077 converted at JP¥ 130 per Eur). Specifically, this result was below the threshold of 5 million yen for the criteria of cost-effectiveness to introduce new interventions in Japan (Fukuda and Shiroiwa, 2019).

At post-intervention, we observed nine responders in the intervention group and two in the control group. Since the intervention cost is as described above, the cost required to increase one responder was calculated to be JP¥ 14,143 (US$ 129, €109). We observed seven participants with remission in the intervention group and two in the control group. Since the intervention cost is as described above, the cost required to increase one patient with remission was calculated to be JP¥ 19,800 (US$ 180, €152).

### 3.4 Therapeutic alliance through process evaluation

At the week 12 time point, the mean of total WAI-SF score of patients who underwent the ICBT was 66.2 (SD = 10.3). This suggests that the therapeutic relationship was well-established in the intervention group.

### 3.5 Secondary analysis

We used the data of the 25 participants who completed the guided ICBT in this RCT and a subsequent rescue trial (shown data details in Supplementary File Table S4). Mean of treatment response rate was 36.7% (SD = 23.8). The results of a multiple regression analysis showed that mild depression (β = −0.518, *t* = −3.09, SE = 0.006, *p* = 0.005) and pharmacotherapy (β = 0.521, *t* = 3.12, SE = 0.082, *p* = 0.005) at baseline predicted changes in obsessive-compulsive symptoms after therapist-guided ICBT (adjusted R^2^ = 0.363; *t* = 5.02, SE = 0.19; *p* < 0.001).

### 3.6 Adverse events

Three adverse events were reported in this RCT. Two were in the intervention group, depressive mood in a female participant and a cold in a male participant. Depressive symptoms of the female patient were controlled by increasing the dose of antidepressants. The participant who had a cold recovered after a few days of rest. In the control group, a female participant was diagnosed with breast cancer. She immediately dropped out this RCT, and focused on cancer treatment.

## 4 Discussion

In this study, we conducted a multicenter RCT to examine the effectiveness of guided ICBT for patients with OCD. The results suggest that guided ICBT in addition to TAU is superior to TAU plus waiting list to ICBT, to improve obsessive-compulsive symptoms.

The results of this RCT support the effectiveness of guided ICBT for OCD. The intervention group showed a significantly greater reduction in obsessive-compulsive symptoms as measured by Y-BOCS and higher rates of response and remission than the control group. This result is consistent with the results of a previous study using the dimensional obsessive-compulsive scale and the obsessional beliefs questionnaire (Mahoney et al., 2014). In this RCT, the effect size between groups was Cohen's *d* = 1.05, which equally high as that of the three previous studies (Andersson et al., 2012; Wootton et al., 2013; Wootton et al., 2019).

This study was first RCT in Japan to investigate effectiveness of guided ICBT for OCD, which extend the findings of a meta-analysis by Wootton (2016). This meta-analysis included studies by telephone-CBT, computerized-CBT, and videoconference-CBT conducted in eighteen countries of the Western world. Considering the results of two pilot trials conducted in South Korea and China (Seol et al., 2016; Zhou et al., 2019), guided ICBT seems to be effective for OCD in the Eastern culture.

The cost-effectiveness analyses showed that guided ICBT may be cost-effective in Japan. Results of this study shows that with the development of ICT, it is possible to offer ICBT effectively at a very low price. Therefore, healthcare policy makers should seriously consider the introduction of ICBT to address the challenge posed by the estimated CBT implementation rate of 6.2% in Japan (Takahashi et al., 2018). Please note that in our RCT, the cost of each participant was not based on observational data. Our findings of the cost-effectiveness should be regard as a preliminary result because of this limitation. The costs can be calculated by using the self-rated Trimbos and Instrument Medical Technology Assessment of Cost Questionnaire for Psychiatry (TIC-P) (Haakkart-van Roijen, 2010). As the TIC-P Japanese version was not available at the beginning of this RCT, we could not collect cost data for each participant. For future research, cost data of each participant should be observed using TIC-P translated to Japanese to analyze more rigorous cost-effectiveness of the intervention in Japan.

We identified two predictors of response to guided ICBT at baseline: low severity of depression and use of antidepressants. This result contradicts the results of Kyrios et al. (2018) that suggested that higher severity of depression suggests a higher improvement of Y-BOCS score. Cognitive impairments are frequently found in patients with major depressive disorder (McIntyre et al., 2015; Millan et al., 2012). Cognitive dysfunction in patients with OCD may impair face-to-face CBT response (Hamatani et al., 2020; Moritz et al., 2005). Therefore, learning cognitive-behavioral skills with guided ICBT may be particularly difficult in patients with OCD who have prominent depressive symptoms. Our results also support the finding that antidepressants when administered in combination with psychotherapy yield better results than monotherapy (Cuijpers et al., 2014; Skapinakis et al., 2016). Knowledge of the factors that improve the benefits of ICBT for patients with OCD is limited (Andersson and Titov, 2014), and therefore, our findings are novel and significant.

This study has the following limitations. Firstly, only the short-term changes in outcomes were evaluated, while the long-term symptomatic improvement was not considered. This should be addressed through future longitudinal studies. We are conducting a two-year follow-up of participants who have completed guided ICBT in this study, and we will hopefully report the results of the survey on the long-term effectiveness of guided ICBT for patients with OCD in the future. Secondly, due to the small sample size, results of the cost-effectiveness from this RCT can be affected by chance. The cost-effective conclusions in this study are therefore preliminary. Future RCTs should be conducted using a larger sample size to investigate cost-effectiveness. Thirdly, we have identified predictors of the patient background that respond to ICBT; however, due to the small sample size, the results need to be careful interpretation.

## 5 Conclusions

This study demonstrated the effectiveness of guided ICBT for patients with OCD. Guided ICBT may be cost-effective for treating OCD in Japan. Furthermore, our secondary analysis yielded preliminary response predictors of response to guided ICBT.

## Declaration of competing interest

The authors declare that they have no known competing financial interests or personal relationships that could have appeared to influence the work reported in this paper.

## Appendix A Supplementary data

Supplementary file. Image 1
