# Supplementary material for: Guided internet-based cognitive behavioral therapy for obsessive-compulsive disorder: A multicenter randomized controlled trial in Japan
Source: Internet Interv. 2022 Feb 24;28:100515. doi: 10.1016/j.invent.2022.100515 (PMC8886053; doi:10.1016/j.invent.2022.100515)
Supplement: Supplementary file 1 — Supplementary file. [file mmc1.docx]

**Supplementary File**

**Guided Internet-Based Cognitive Behavioral Therapy for Obsessive-Compulsive Disorder: A Multicenter Randomized Controlled Trial in Japan**

Table S1. Changes of the Protocol for this Randomized Controlled Trial and Reasons

| Items | Change | Reasons |
| --- | --- | --- |
| Research facilities | Added Kokoro to Karada Clinic Fukui has both psychiatry and psychosomatic medicine. | We added a facility during this trial to recruit participants enough to meet the required sample size. |
| Sample size | The sample size has been changed to require 32 participants from 30. | Because the estimated dropout rate for each group has been revised from 5% to 10%. |
| Outcome | Replaced the Beck Anxiety Inventory† with the Obsessive-Compulsive Inventory (OCI) on a secondary outcome. | To assist in understanding the primary outcome, added OCI to assess obsessive-compulsive symptoms. |
| Statistical analyses | Full analysis set was created excluding data of a participant conducted no session in the intervention group. In other words, we have withdrawn from adopting the intention-to-treatment principle. | Because analyses are performed by excluding the minimum exemptible subjects from all the subjects. |

*Note.* *All changes have been approved by the Institutional Review Board in Chiba University Hospital.*

†Beck, A. T., Epstein, N., Brown, G., Steert, R. A., 1988. An inventory for measuring clinical anxiety: psychometric properties. Journal of consulting and clinical psychology, 56(6), 893–897. <https://doi.org/10.1037//0022-006x.56.6.893>

Table S2. CONSORT 2010 checklist of information to include when reporting a randomised trial

| Section/Topic | Item No | Checklist item | Reported on page No |
| --- | --- | --- | --- |
| Title and abstract | | | |
|  | 1a | Identification as a randomised trial in the title | Page 3. |
|  | 1b | Structured summary of trial design, methods, results, and conclusions (for specific guidance see CONSORT for abstracts) | Page 3. |
| Introduction | | | |
| Background and objectives | 2a | Scientific background and explanation of rationale | Pages 4-5. |
|  | 2b | Specific objectives or hypotheses | Page 6. |
| Methods | | | |
| Trial design | 3a | Description of trial design (such as parallel, factorial) including allocation ratio | Pages 5-6. |
|  | 3b | Important changes to methods after trial commencement (such as eligibility criteria), with reasons | Page 6. |
| Participants | 4a | Eligibility criteria for participants | Page 8. |
|  | 4b | Settings and locations where the data were collected | Page 11. |
| Interventions | 5 | The interventions for each group with sufficient details to allow replication, including how and when they were actually administered | Pages 8-11. |
| Outcomes | 6a | Completely defined pre-specified primary and secondary outcome measures, including how and when they were assessed | Pages 10-12. |
|  | 6b | Any changes to trial outcomes after the trial commenced, with reasons |  |
| Sample size | 7a | How sample size was determined | Page 6. |
|  | 7b | When applicable, explanation of any interim analyses and stopping guidelines | N/A |
| Randomisation: |  |  | Page 8. |
| Sequence generation | 8a | Method used to generate the random allocation sequence | Page 8. |
|  | 8b | Type of randomisation; details of any restriction (such as blocking and block size) | Page 8. |
| Allocation concealment mechanism | 9 | Mechanism used to implement the random allocation sequence (such as sequentially numbered containers), describing any steps taken to conceal the sequence until interventions were assigned | Page 8. |
| Implementation | 10 | Who generated the random allocation sequence, who enrolled participants, and who assigned participants to interventions | Page 8. |
| Blinding | 11a | If done, who was blinded after assignment to interventions (for example, participants, care providers, those assessing outcomes) and how | Page 13. |
|  | 11b | If relevant, description of the similarity of interventions | - |
| Statistical methods | 12a | Statistical methods used to compare groups for primary and secondary outcomes | Pages 12-13. |
|  | 12b | Methods for additional analyses, such as subgroup analyses and adjusted analyses | Pages 14-15. |
| Results | | | |
| Participant flow (a diagram is strongly recommended) | 13a | For each group, the numbers of participants who were randomly assigned, received intended treatment, and were analysed for the primary outcome | Page 7. |
|  | 13b | For each group, losses and exclusions after randomisation, together with reasons | Page 15. |
| Recruitment | 14a | Dates defining the periods of recruitment and follow-up | Pages 6-7. |
|  | 14b | Why the trial ended or was stopped | - |
| Baseline data | 15 | A table showing baseline demographic and clinical characteristics for each group | Page 15. |
| Numbers analysed | 16 | For each group, number of participants (denominator) included in each analysis and whether the analysis was by original assigned groups | Page 15. |
| Outcomes and estimation | 17a | For each primary and secondary outcome, results for each group, and the estimated effect size and its precision (such as 95% confidence interval) | Pages 16-17. |
|  | 17b | For binary outcomes, presentation of both absolute and relative effect sizes is recommended | - |
| Ancillary analyses | 18 | Results of any other analyses performed, including subgroup analyses and adjusted analyses, distinguishing pre-specified from exploratory | Pages 18-19. |
| Harms | 19 | All important harms or unintended effects in each group (for specific guidance see CONSORT for harms) | Page 19. |
| Discussion | | | |
| Limitations | 20 | Trial limitations, addressing sources of potential bias, imprecision, and, if relevant, multiplicity of analyses | Page 21. |
| Generalisability | 21 | Generalisability (external validity, applicability) of the trial findings | Page 21. |
| Interpretation | 22 | Interpretation consistent with results, balancing benefits and harms, and considering other relevant evidence | Pages 20-21. |
| Other information | | |  |
| Registration | 23 | Registration number and name of trial registry | Page 5. |
| Protocol | 24 | Where the full trial protocol can be accessed, if available | Page 5. |
| Funding | 25 | Sources of funding and other support (such as supply of drugs), role of funders | Pages 21-22. |

**Summary of a Rescue Trial for Participants Allocated the Control Group**

We reported summary of a rescue trial here. At baseline, two participants were in remission, one dropped out of the previous RCT, and one declined. Hence, 12 people participated. Procedures for the intervention and outcomes were conducted in similar methods to the previous RCT. This trial was approved by the ethics committee (G2019027) and registered in the Japanese clinical trial registration database (UMIN000040699). The measured symptom outcomes were statistically analyzed using a paired *t*-test to investigate changes in clinical symptoms. *P*-value criteria and statistical analysis tools followed the same manors as the preceding RCT. We excluded data of a female participant reported that she conducted no session. In 11 participants included in the analyses, means were 29.3 years old (SD = 12.3), estimated IQ 102.2 (5.8), and seven female (63.6%). The toral Y-BOCS scores (range, 0–40) significantly decreased (*p* < 0.001) within the intervention group from a mean (SD) of 23.4 (5.2) to 14.5 (6.3), with an effect size of Cohen's *d* = 1.51. The total OCI scores (range, 0–168) significantly decreased (*p* = 0.002) from a mean (SD) of 63.6 (22.6) to 37.0 (20.3), with an effect size of Cohen's *d* = 1.28. The total PHQ-9 scores (range, 0–27) significantly decreased (*p* = 0.004) from a mean (SD) of 11.9 (5.5) to 8.4 (4.6), with an effect size of Cohen's *d* = 1.11. The total GAD-7 scores (range, 0–21) significantly decreased (*p* = 0.007) from a mean (SD) of 10.9 (3.2) to 6.4 (4.6), with an effect size of Cohen's *d* = 1.01. Quality-adjusted life years estimated by using EQ-5D (range, 0–1.0000) significantly increased (*p* = 0.003) from a mean (SD) of 0.6629 (0.1534) to 0.7786 (0.1193), with an effect size of Cohen's *d* = 0.77. Rates of treatment response and remission were 81.2% (n = 9/11) and 45.5% (n = 5/11), respectively. The mean of total WAI-SF score was 71.6 (SD = 10.4) at post-intervention.

**Table S3. Results of Blinding Assessment by Two Independent Assessors in this Randomized Controlled Trial**

|  | Assumed Intervention | Assumed Control | I don’t know | Total |
| --- | --- | --- | --- | --- |
| Intervention group  (n = 14) | 7 | 4 | 3 | 14 |
| Control group  (n = 15) | 5 | 6 | 4 | 15 |
| Total  (n = 29) | 12 | 10 | 7 | 29 |

**Table S4. Character of 25 participants completed the intervention**

| Items | Data at baseline  (n=25) |
| --- | --- |
| Sex (Female), n | 15 (60%) |
| Pharmacotherapy‡, n | 15 (60%) |
| Estimated IQ (JART), mean (SD) | 102.2 (10.0) |
| Obsessive-compulsive symptoms (OCD), mean (SD) | 22.9 (4.6) |
| Depression (PHQ-9), mean (SD) | 11.8 (6.7) |
| Generalized anxiety (GAD-9), mean (SD) | 10.9 (5.6) |

GAD, generalized anxiety disorder; JART, Japanese adult rating test; PHQ, patient health questionnaire; Y-BOCS, Yale-Brown obsessive-compulsive scale

‡Described the generic name of antidepressant used in pharmacotherapy (n): Escitalopram Oxalate (6), Paroxetine Hydrochloride Hydrate (5), Fluvoxamine Maleate (2), Clomipramine Hydrocholoride (3), Duloxetine Hydrochloride (2), Trazodone Hydrochloride (1). Three participants were taking two antidepressants.
